# Wogonin prevents TLR4-NF-κB-medicated neuro-inflammation and improves retinal ganglion cells survival in retina after optic nerve crush

**DOI:** 10.18632/oncotarget.12700

**Published:** 2016-10-16

**Authors:** Yue Xu, Boyu Yang, Yaguang Hu, Lin Lu, Xi Lu, Jiawei Wang, Fan Xu, Shanshan Yu, Jingjing Huang, Xiaoling Liang

**Affiliations:** ^1^ State Key Laboratory of Ophthalmology, Zhongshan Ophthalmic Center, Sun Yat-sen University, Guangzhou, Guangdong, People's Republic of China

**Keywords:** wogonin, glaucoma, TLR4-NF-κB pathways, retinal ganglion cells, inflammatory responses, Pathology Section

## Abstract

Chronic neuro-inflammation is involved in the death of retinal ganglion cells (RGCs) in glaucoma. The aim of this study is to determine whether wogonin can suppress inflammatory responses and rescue RGCs death after optic nerve crush (ONC), an ideal animal model of glaucoma. Wogonin was administered intraperitoneally 10 min after establishment of ONC model. In this study, wogonin treatment reduced RGCs loss and inhibited RGCs apoptosis demonstrated by the increased Brn3a labeling RGCs at day 14 and the decreased cleaved caspase-3 expression at day 7 after ONC, respectively. In ONC model, number of GFAP-positive glial cells and iba1-positive microglial cells were increased, combined of the elevated level of pro-inflammatory cytokines released in retina at day 7. However, most of these responses were inhibited after wogonin treatment. The level of TLR4 expression, NF-κB-P65 nucleus location and NF-κB-P65 phosphorylation were increased in retina at day 1 after ONC, which was significantly reduced after wogonin treatment. These results demonstrated that wogonin protected RGCs survival and suppressed neuro-inflammation in retina after ONC by inhibiting TLR4-NF-κB pathways. We conclude that wogonin could be a possible strategy for the treatment of glaucoma.

## INTRODUCTION

Glaucoma, a family of neurodegenerative diseases, is the second leading cause of blindness worldwide [1]. It was estimated that 8.4 million people suffering from blindness caused by glaucoma in 2010 alone, and the number will rise to 79.6 million by the year of 2020 [2, 3]. Glaucoma is considered a progressive neurodegenerative eye disorder characterized by the loss of retinal ganglion cells (RGCs), ultimately leading to visual field loss [4-6]. However, treatment options for patients with glaucoma remain limited and minimally efficacious [3, 5]. Though much work has devoted to explore the neuro-protective therapies to attenuate axotomy-induced RGCs death [4, 5], there are no practically effective drugs to date [5]. Therefore, it is currently of considerable interest to develop effective neuro-protective treatment strategies to preserve or regenerate RGCs in glaucoma.

Numbers of mechanisms have been linked to axotomy-induced RGCs death in glaucoma [7-9]. A leading theory is that the chronic neuro-inflammation generated by the activation of neuroglial cells (microglia, astrocytes, and Müller cells) which is involved in a number of complex signaling pathways [10, 11] in the retina after glaucoma [7, 8]. Recent studies have shown that activated neuroglial cells dramatically induced the activation of toll-like receptor 4-nuclear factor-kappa B (TLR4-NF-κB) pathways, which may play a vital role in promoting the release of pro-inflammatory cytokines, causing RGCs death in retina after optic nerve (ON) injury [11, 12]. There was evidence to show a reduced inflammatory responses and improved RGCs preservation following optic nerve crush (ONC) in TLR4-deficient mice, which indicated that the presence of TLR4-dependent pathway induced the RGCs death [13]. Furthermore, reducing TLR4-NF-κB pathways dependent glial activation with medical treatment could promote RGCs survival after ON injury [14, 15]. These studies suggested that pharmacological inhibition of TLR4-NF-κB pathways might be an effective neuro-protective treatment strategy for the protection of RGCs death in retina after injury.

Wogonin (5,7-dihydroxy-8-methoxyflavone), one of the major constituents of *Scutellaria baicalensis Georgi* (also called *Huang-Qin*), has been widely used in treating various diseases including allergy [16], tumors [17], inflammatory and neurological diseases [18, 19]. Experimental studies *in vitro* have demonstrated that wogonin could reduce endoplasmic reticulum (ER) stress and apoptosis in Tunicamycin-induced rat dorsal root ganglion (DRG) neurons [20, 21], and suppress lipopolysaccharide (LPS)-induced release of pro-inflammatory cytokines in rat DRG neurons by inhibiting TLR4-NF-κB pathways [22]. In addition, wogonin could suppress LPS-induced release of pro-inflammatory cytokines [23, 24] and reduce migration [25] of microglial cells by inhibiting NF-κB-dependent pathways. Moreover, the neuro-protective effect of wogonin has also been demonstrated in different central nervous system (CNS) ischemia animal models. Recent study has demonstrated that wogonin could reduce infarct volume and inhibit ischemic brain injury against permanent middle cerebral artery occlusion in a rat model [26]. In addition, wogonin could significantly reduce TLR4-NF-κB pathways-induced activation of inflammatory responses and improve histological and functional outcomes in an animal model of traumatic brain injury (TBI) [27]. These evidences showed that the neuro-protective effects of wogonin were considered to be due in part to its anti-inflammatory responses in microglial cells *via* TLR4-NF-κB pathways. However, there is a lack of data describing the neuro-protective effect of wogonin in retina after injury.

In our present study, we firstly investigated the protective effects of wogonin on RGCs death, inflammatory responses, and neuroglial activation in retina after ONC, an ideal animal model of glaucoma. We further examined whether these anti-inflammatory and neuro-protective effects were through inhibiting ONC-induced activation of the TLR4-NF-κB pathways.

## RESULTS

### Wogonin reduced loss of RGCs in retina after ONC

**Figure 1:**
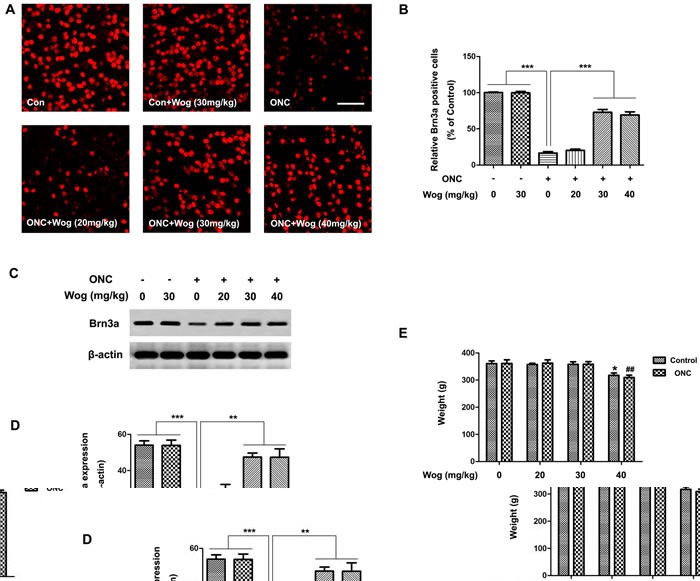
The effect of wogonin on loss of RGCs in the retina after ONC Rats retinas from different groups were harvested at day 14 after ONC and subjected to whole mount immunostaining and western blot analysis with Brn3a. **A.** Representative images from control group, wogonin group, ONC group, ONC-plus-20 mg/kg wogonin group, ONC-plus-30 mg/kg wogonin group, ONC-plus-40 mg/kg wogonin group were stained by Brn3a (red). Scale bar = 100 μm. **B.** Densitometric analysis of effects of wogonin on the survival of the RGCs in the GCL, which was assessed by counting the number of fluorescent Brn3a stained RGCs. Data were shown as mean ± SEM (*n* = 6 per group, ***p < 0.001). **C.** The protein level of Brn3a was evaluated by western blot analysis. β-actin was used to ensure equal loading. **D.** Densitometric analysis of effects of wogonin on protein expression of Brn3a. Data were shown as mean ± SEM (*n* = 6 per group, ***p < 0.001, **p < 0.01). **E.** The body weight was measured at day 14 after wogonin intraperitoneally injection at different concentrations. Data were shown as mean ± SEM (*n* = 6 per group, *p < 0.05 comparing 40 mg/kg wogonin group *versus* control group, ^##^*p* < 0.01 comparing ONC-plus-40 mg/kg wogonin group *versus* ONC group).

The survival of the RGCs was evaluated by Brn3a staining on the retinal flat mounting at day 14 after ONC. As shown in Figure 1A-1B, the number of Brn3a-stained RGCs decreased after ONC (***p < 0.001). Wogonin had no effect on the density of RGCs in normal control. More Brn3a-stained RGCs were observed in the ONC-plus-wogonin group as compared with the ONC group (***p < 0.001). In addition, western blot data in Figure 1C-1D also showed a marked decreased expression of Brn3a protein in ONC group (***p < 0.001), which was increased in ONC-plus-wogonin group (**p < 0.01). Moreover, the protection effect of wogonin seemed not obvious in the concentration of 20 mg/kg, but turned out to be significant in the concentration of 30 mg/kg (**p < 0.01). The neuro-protective effect of 30 mg/kg and 40 mg/kg wogonin were statistically equal, while the body weight of rats decreased after intraperitoneal injection of 40 mg/kg wogonin (*p < 0.05, ^##^*p* < 0.01,) (Figure 1E). Thus, 30 mg/kg wogonin was chosen in the subsequent experiments.

### Wogonin inhibited apoptosis of RGCs in retina after ONC

**Figure 2:**
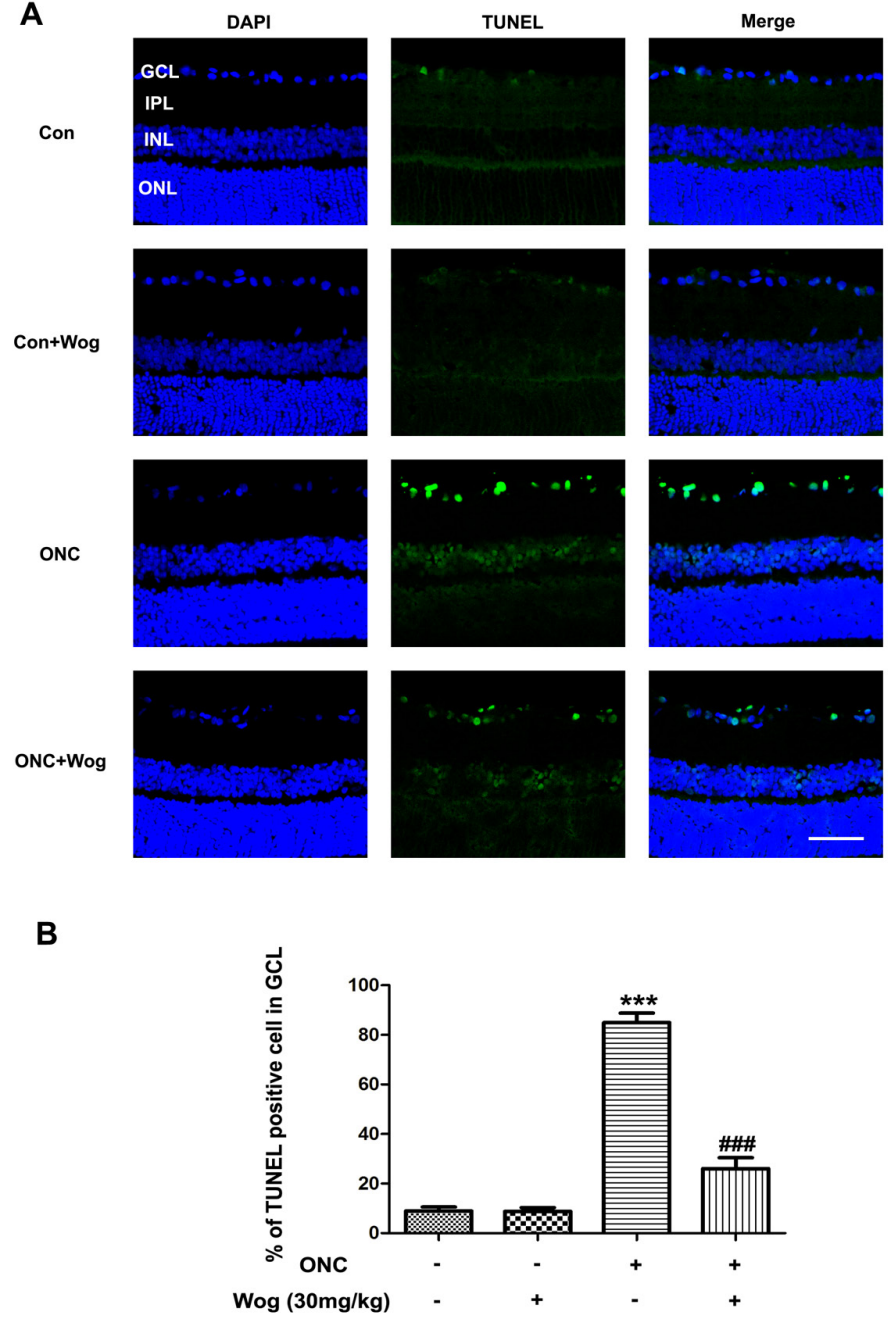
The effect of wogonin on the density of TUNEL-positive RGCs in the retina after ONC Rats retinas from different groups were harvested at day 7 after ONC and subjected to frozen sections immunostaining with TUNEL. **A.** Rats retinas from control group, wogonin group, ONC group, ONC-plus-wogonin group were stained by TUNEL (green) and DAPI (blue). Scale bar = 100 μm. **B.** Densitometric analysis of effects of wogonin on the apoptotic ratio of the RGCs in the GCL, which was assessed by counting the number of TUNEL-positive RGCs. Data were shown as mean ± SEM (*n* = 6 per group, ***p < 0.001 comparing ONC group *versus* control group, ^###^*p* < 0.001 comparing ONC-plus-wogonin group *versus* ONC group). Abbreviations: GCL, ganglion cell layer; INL, inner nuclear layer; IPL, inner plexiform layer; ONL, outer nuclear layer.

To further evaluate the anti-apoptotic effects of wogonin, we used terminal-deoxynucleotidyl transferase mediated nick end labeling (TUNEL) staining to explore whether wogonin could reduce ONC-induced RGCs apoptosis. As shown in Figure 2A, control group had fewer TUNEL-positive cells in retina. Wogonin had no effect on the density of TUNEL-positive cells in normal control. There were notably more TUNEL-positive cells in the retina after ONC, most of which were located in ganglion cell layer (GCL) (***p < 0.001). In addition, fewer TUNEL-positive cells were observed in the ONC-plus-wogonin group as compared with the ONC group.

### Wogonin attenuated the activation of caspase-3 in retina after ONC

**Figure 3:**
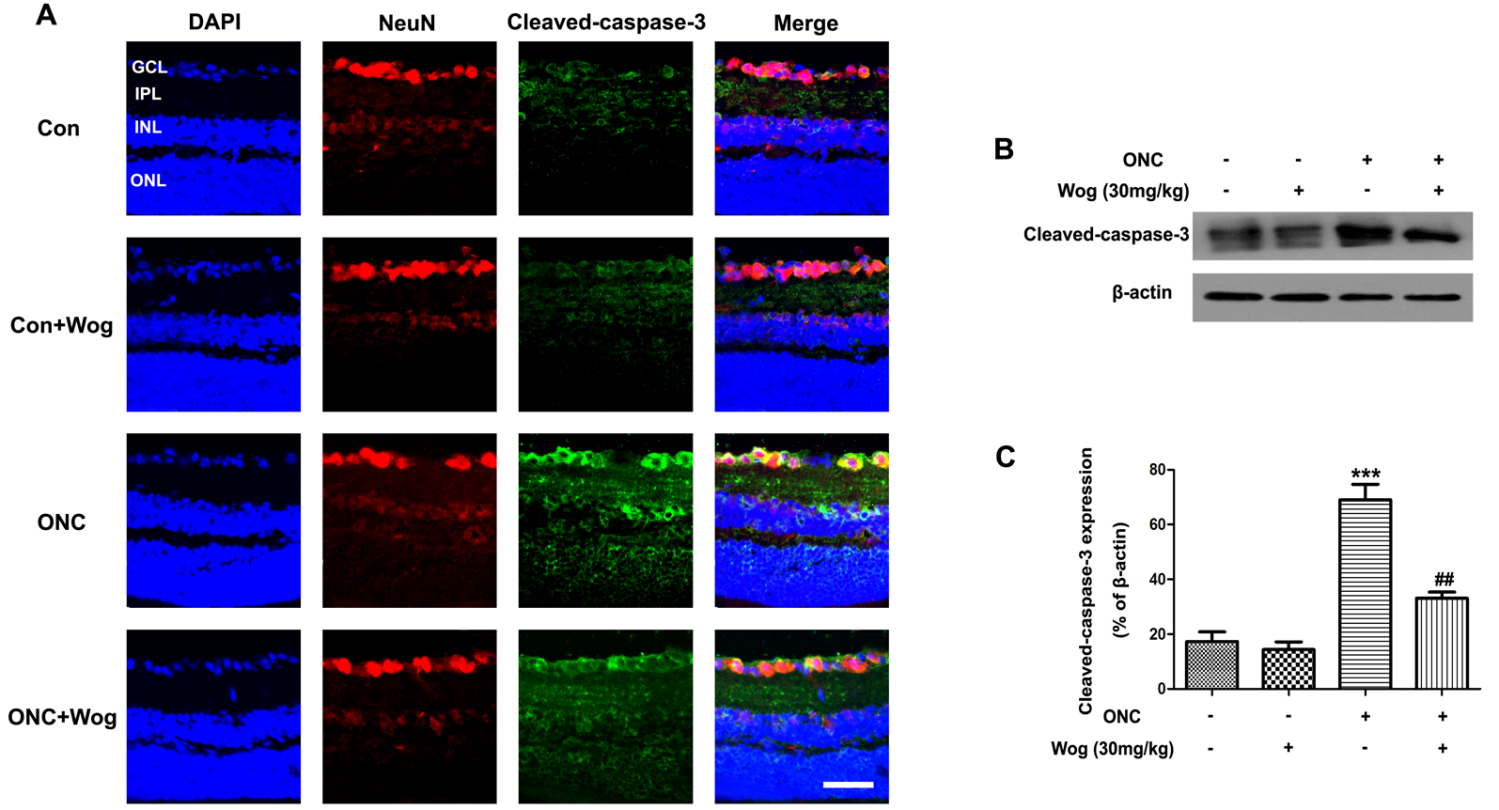
The effect of wogonin on the activity of caspase-3 in the retina after ONC Rats retinas from different groups were harvested at day 7 after ONC and subjected to frozen sections immunostaining and western blot analysis with cleaved-caspase-3. **A.** Rats retinas from control group, wogonin group, ONC group, ONC-plus-wogonin group were stained by NeuN (red), cleaved-caspase-3 (green) and DAPI (blue). Scale bar = 100 μm. **B.** The protein level of cleaved-caspase-3 was evaluated by western blot analysis. β-actin was used to ensure equal loading. **C.** Densitometric analysis of effects of wogonin on protein expression of cleaved-caspase-3. Data were shown as mean ± SEM (*n* = 6 per group, ***p < 0.001 comparing ONC group *versus* control group, ^##^*p* < 0.01 comparing ONC-plus-wogonin group *versus* ONC group). Abbreviations: GCL, ganglion cell layer; INL, inner nuclear layer; IPL, inner plexiform layer; ONL, outer nuclear layer.

As mentioned in previous study [28], caspase-3 activity was involved in the regulation of RGCs apoptosis. Therefore, to further explore the association between wogonin and RGCs protection, we firstly examined the expression of cleaved-caspase-3 at day 7 after ONC by western blot (Figure 3B-3C). After ONC, the expression of cleaved-caspase-3 was up-regulated (***p < 0.001), which was attenuated in ONC-plus-wogonin group (^##^*p* < 0.01). Furthermore, immunofluorescent staining results were consistent with that in western blot, showing that wogonin inhibited the cleaved-caspase-3 expression in retina after ONC (Figure 3A). In addition, the co-localization of increased cleaved-caspase-3 and Neuronal nuclei (NeuN) were detected in RGCs at day 7 after ONC (Figure 3A). All together, these results indicated that wogonin might attenuate RGCs apoptosis in a caspase-3-dependent way after ONC.

### Wogonin attenuated the gliosis responses in retina after ONC

**Figure 4:**
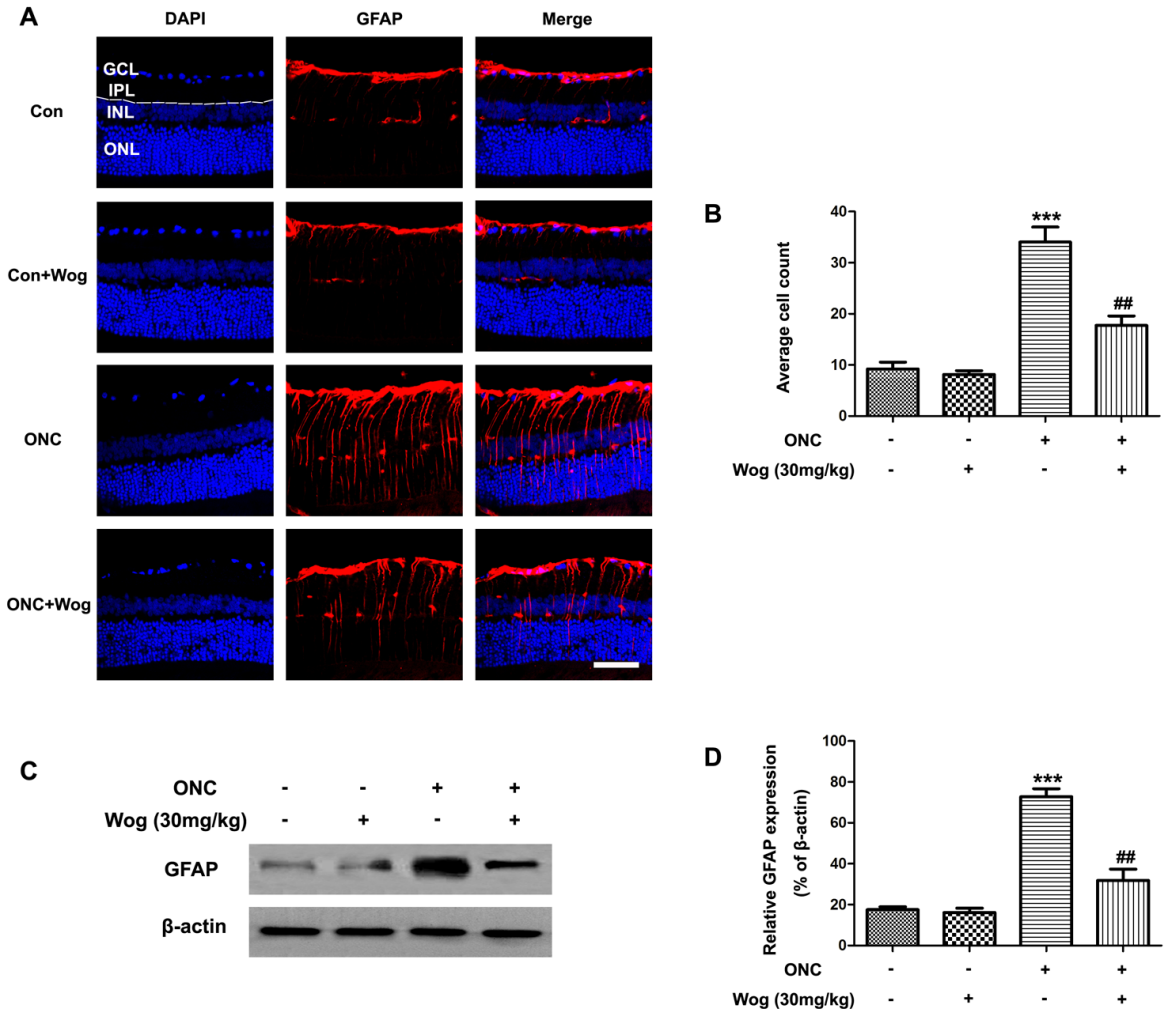
The effect of wogonin on the number of GFAP-positive fibers in the IPL of retina after ONC Rats retinas from different groups were harvested at day 7 after ONC and subjected to frozen sections immunostaining and western blot analysis with GFAP. **A.** Rats retinas from control group, wogonin group, ONC group, ONC-plus-wogonin group were stained by GFAP (red) and DAPI (blue). Scale bar = 100 μm. **B.** Densitometric analysis of effects of wogonin on the glial dysfunction, which was assessed by counting the number of GFAP-positive fibers in the IPL. Data were shown as mean ± SEM (*n* = 6 per group, ***p < 0.001 comparing ONC group *versus* control group, ^##^*p* < 0.01 comparing ONC-plus-wogonin group *versus* ONC group). **C.** The protein level of GFAP was evaluated by western blot analysis. β-actin was used to ensure equal loading. **D.** Densitometric analysis of effects of wogonin on protein expression of GFAP. Data were shown as mean ± SEM (*n* = 6 per group, ***p < 0.001 comparing ONC group *versus* control group, ^##^*p* < 0.01 comparing ONC-plus-wogonin group *versus* ONC group). Abbreviations: GCL, ganglion cell layer; INL, inner nuclear layer; IPL, inner plexiform layer; ONL, outer nuclear layer.

In normal retina, typical glial fibrillary acidic protein (GFAP) staining pattern was localized to a population of astrocytes and retinal Müller cells (Figure 2A). ONC induced a strong up-regulation of GFAP in both astrocytes and Müller cells at day 7 (Figure 2A-2B) (***p < 0.001). Wogonin treatment significantly decreased the number of GFAP-positive fibers in inner plexiform layer (IPL) (##*p* < 0.01) (Figure 2A and 2B). Furthermore, western blot results were consistent with that in immunofluorescent staining, showing that wogonin inhibited the gliosis responses in astrocytes and Müller cells of retina after ONC (***p < 0.001, ^##^*p* < 0.01) (Figure 4C-4D).

### Wogonin inhibited microglial activation in retina after ONC

**Figure 5:**
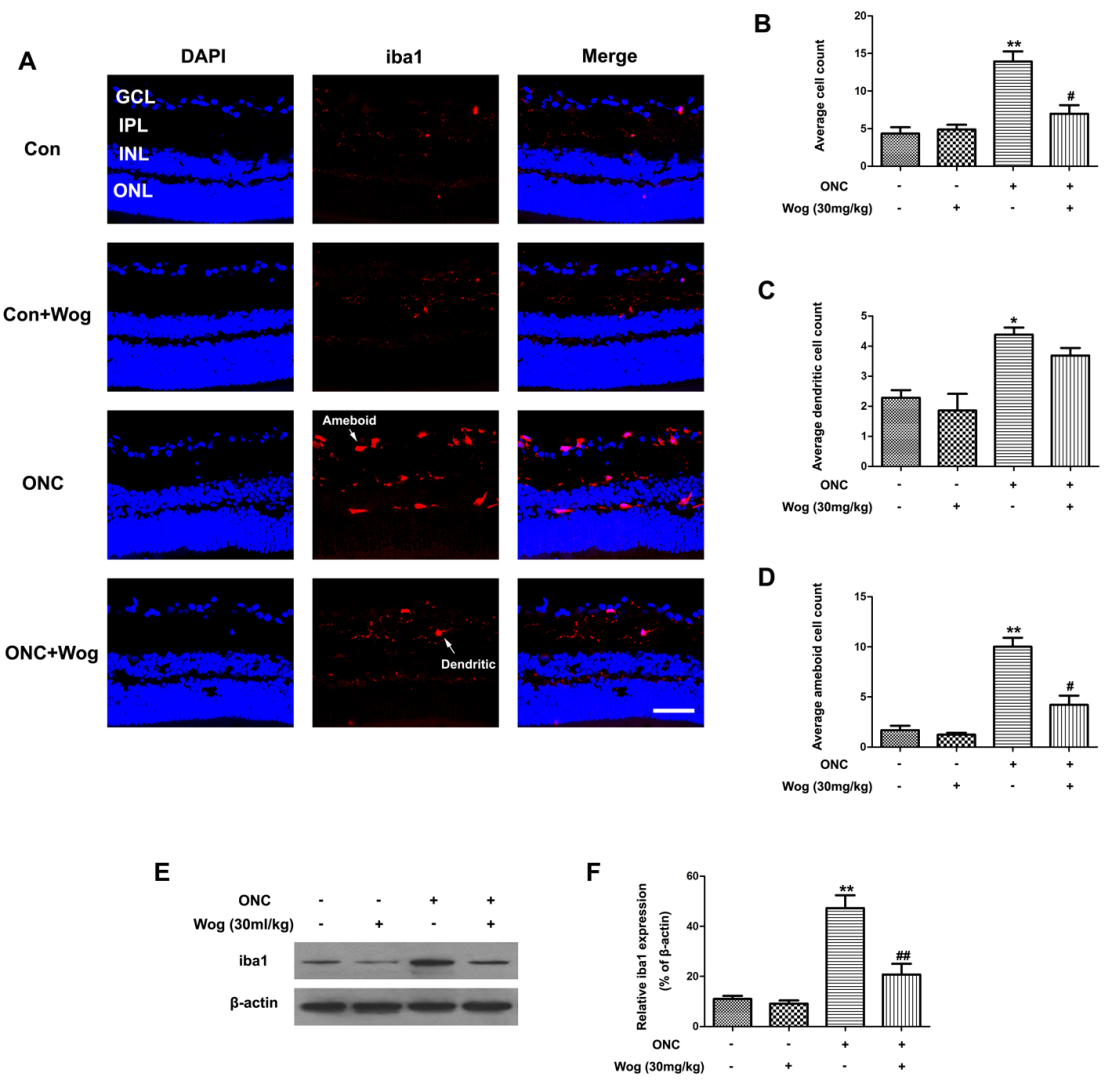
The effect of wogonin on the microglial activation in the retina after ONC Rats retinas from different groups were harvested at day 7 after ONC and subjected to frozen sections immunostaining and western blot analysis with iba1. **A.** Rats retinas from control group, wogonin group, ONC group and ONC-plus-wogonin group were stained by iba1 (red) and DAPI (blue). Scale bar = 100 μm. **B.**-**D.** Densitometric analysis of effects of wogonin on the microglial activation, which was assessed by counting the number of iba1-positive overall microglia numbers **B.**, microglia with a dendritic phenotype **C.** or amoeboid cells **D.**. Data were shown as mean ± SEM (*n* = 6 per group, *p < 0.05 **p < 0.01 comparing ONC group *versus* control group, ^#^*p* < 0.05 comparing ONC-plus-wogonin group *versus* ONC group). **E.** The protein level of iba1 was evaluated by western blot analysis. β-actin was used to ensure equal loading. **F.** Densitometric analysis of effects of wogonin on protein expression of iba1. Data were shown as mean ± SEM (*n* = 6 per group, **p < 0.01 comparing ONC group *versus* control group, ^##^*p* < 0.01 comparing ONC-plus-wogonin group *versus* ONC group). Abbreviations: GCL, ganglion cell layer; INL, inner nuclear layer; IPL, inner plexiform layer; ONL, outer nuclear layer.

As shown in the immunofluorescent staining of retinal sections, ONC could induce an increase in ilonized calcium-binding adapter molecule 1 (iba1)-positive microglial cells in GCL and IPL at day 7 (Figure 5A). In addition, we also detected a significant shift in non-activated, dendritic phenotype toward activated, amoeboid cells (Figure 5C-5D). Wogonin not only decreased overall microglial cells numbers, but also significantly increased the proportion of dendritic cells and decreased the proportion of amoeboid cells when compared with ONC group (*p < 0.05, **p < 0.01, ^#^*p* < 0.05) (Figure 5B-5D). Furthermore, western blot results showed that wogonin treatment significantly prevented iba1 protein expression in retina after ONC (**p < 0.01, ^##^*p* < 0.01) (Figure 5E-5F). These results indicated that wogonin could inhibit the microglial activation in retina after ONC (***p < 0.001, ^##^*p* < 0.01) (Figure 5C-5D).

### Wogonin inhibited pro-inflammatory cytokines expression in retina after ONC

**Figure 6:**
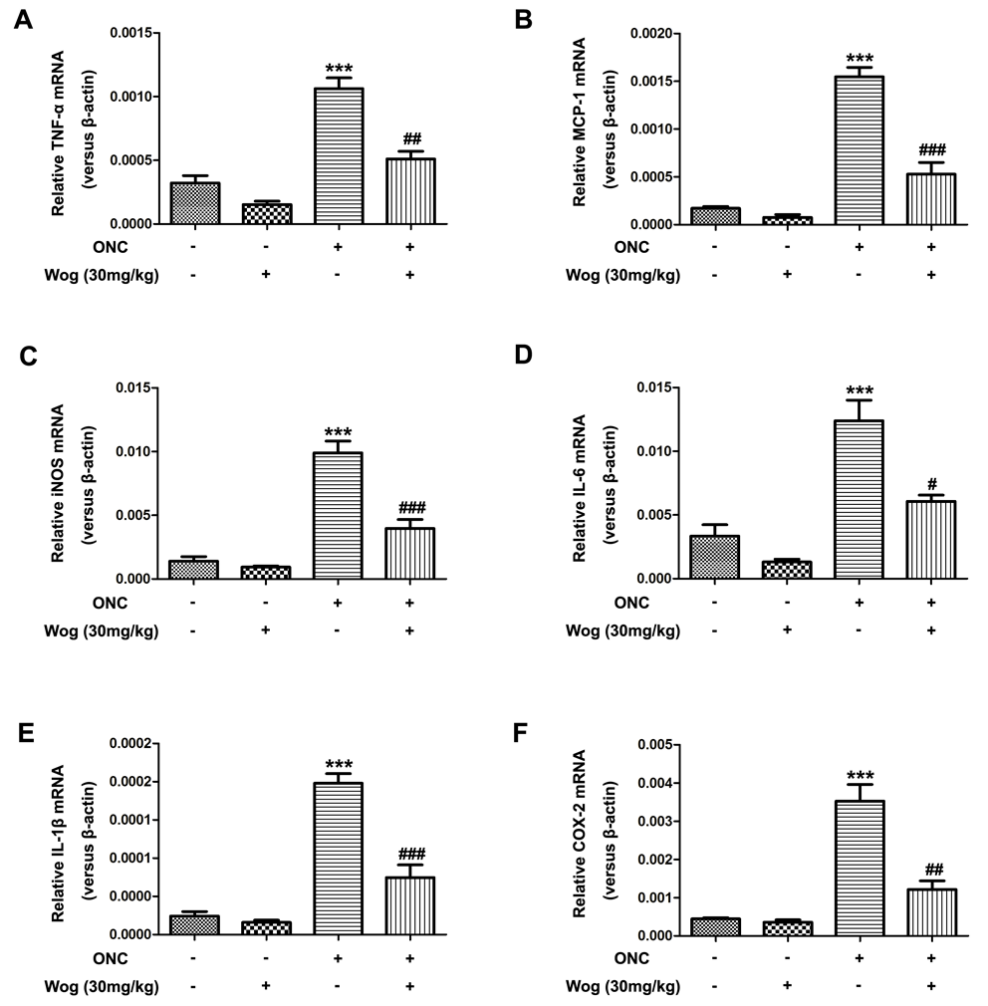
The effect of wogonin on the release of pro-inflammatory cytokines in the retina after ONC Rats retinas from different groups were harvested at day 7 after ONC and subjected to real time PCR analysis. The mRNA levels of TNF-α (Figure 6A), MCP-1 (Figure 6B), iNOS (Figure 6C), IL-6 (Figure 6D), IL-1β (Figure 6E), and COX-2 (Figure 6F) genes in the retina from control group, wogonin group, ONC group and ONC-plus-wogonin group were examined quantitatively by real time PCR. Data were shown as mean ± SEM (*n* = 6 per group, ***p < 0.001 comparing ONC group *versus* control group, ^#^*p* < 0.05, ^##^*p* < 0.01, ^###^*p* < 0.001 comparing ONC-plus-wogonin group *versus* ONC group).

Next, we investigated the effects of wogonin on the release of pro-inflammatory cytokines in retina at day 7 after ONC. The mRNA expressions of tumor necrosis factor-α (TNF-α) (Figure 6A), monocyte chemoat-tractant protein-1 (MCP-1) (Figure 6B), inducible nitric oxide synthase (iNOS) (Figure 6C), interleukin (IL)-6 (Figure 6D), IL-1β (Figure 6E), and cyclooxygenase-2 (COX-2) (Figure 6F) genes in the retina were examined quantitatively by real time polymerase chain reaction (real time PCR). Our results showed that these pro-inflammatory cytokines increased at day 7 after ONC (***p < 0.001), which were down-regulated by wogonin treatment (^#^*p* < 0.001, ^##^*p* < 0.001, ^###^*p* < 0.001) (Figure 6). In addition, the changes of the release of these pro-inflammatory cytokines in retina that treated with wogonin were not obvious compare to those in control group.

### Wogonin down-regulated the activation of TLR4-NF-κB pathways in the retina after ONC

**Figure 7:**
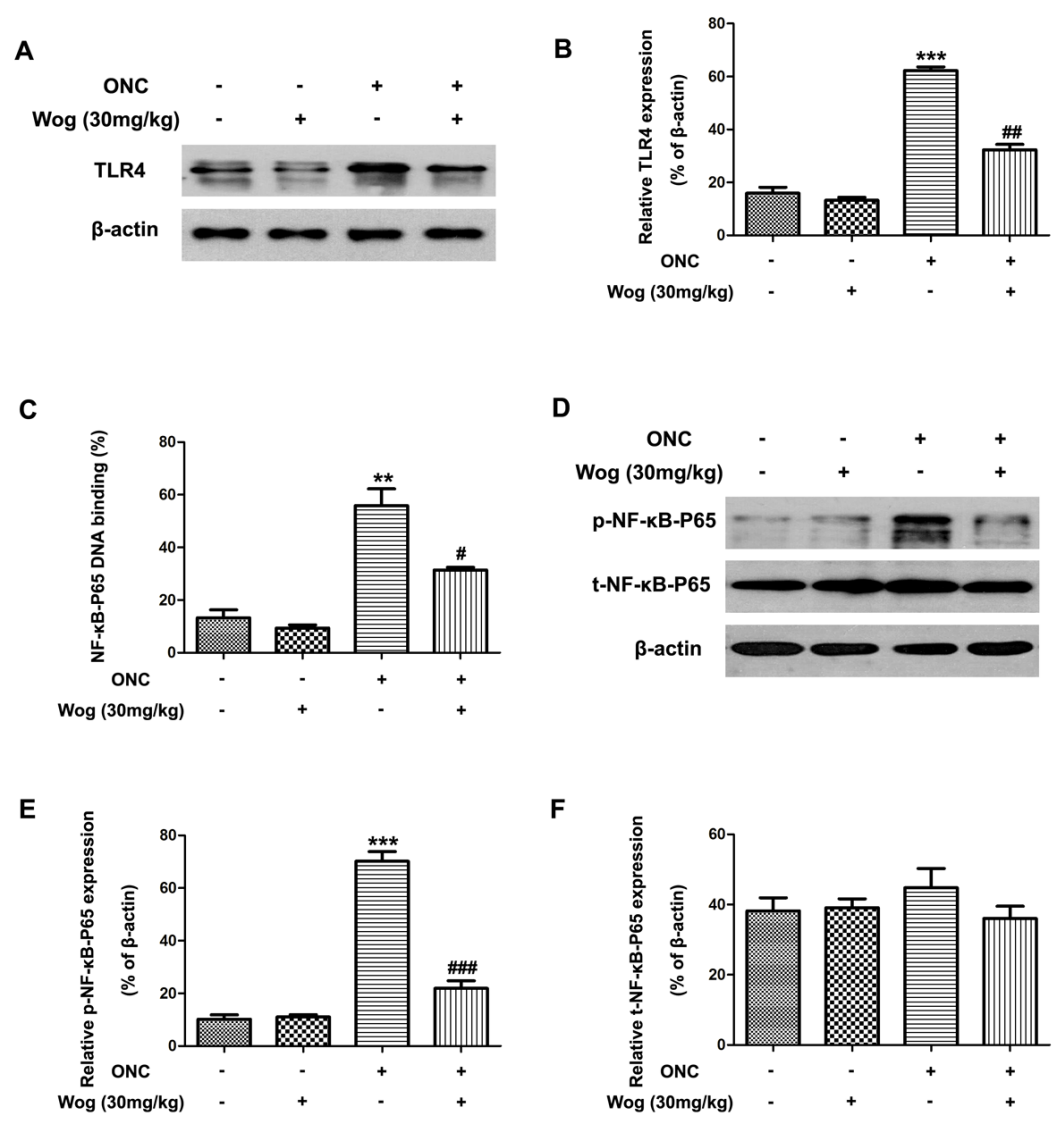
The effect of wogonin on the activation of TLR4-NF-κB pathways in the retina after ONC Rats retinas from different groups were harvested at day 1 after ONC and subjected to western blot analysis. **A.** The protein level of TLR4 in the retina from control group, wogonin group, ONC group and ONC-plus-wogonin group were examined by western blot analysis. β-actin was used to ensure equal loading. **B.** Densitometric analysis of effects of wogonin on protein expression of TLR4. Data were shown as mean ± SEM (*n* = 6 per group, ***p < 0.001 comparing ONC group *versus* control group, ^##^*p* < 0.01 comparing ONC-plus-wogonin group *versus* ONC group). **C.** Nuclear extracts were prepared by using a nuclear extract kit. NF-κB-P65 activity was measured using an ELISA kit. Data were shown as mean ± SEM (*n* = 6 per group, **p < 0.01 comparing ONC group *versus* control group, ^#^*p* < 0.05 comparing ONC-plus-wogonin group *versus* ONC group). **D.** The protein level of p-NF-κB-P65 and t-NF-κB-P65 in the retina from control group, wogonin group, ONC group and ONC-plus-wogonin group were examined by western blot analysis. β-actin was used to ensure equal loading. **E.-F.** Densitometric analysis of effects of wogonin on protein expression of p-NF-κB-P65 and t-NF-κB-P65. Data were shown as mean ± SEM (*n* = 6 per group, ***p < 0.001 comparing ONC group *versus* control group, ^###^*p* < 0.001 comparing ONC-plus-wogonin group *versus* ONC group). Abbreviations: p, phosphorylated; t, total.

Previous studies have shown that the TLR4-NF-κB pathways were involved in inflammatory responses and microglial activation, which were associated with RGCs death [29, 30]. Thus, we investigated whether wogonin could down-regulated TLR4-NF-κB pathways in retina on day 1 after ONC. In Figure 7A-7B, western blot results showed the protein expression of TLR4 increased in retina at day 1 after ONC (***p < 0.001), which was reduced after treatment with wogonin (^##^*p* < 0.01), suggesting the significant inhibitory effect of wogonin on increased TLR4 expression in retina on day 1 after ONC.

In addition, our results also showed that the ratio of NF-κB-P65 DNA-binding dramatically increased on day 1 after ONC (**p < 0.01), which was reduced by treatment with wogonin (^#^*p* < 0.05) (Figure 7C). Moreover, ONC caused increase in phosphorylated (p)-NF-κB-P65 from the control level (***p < 0.001), which was significantly suppressed by wogonin treatment (^###^*p* < 0.001) (Figure 7D-7E). However, there was no obvious change in total (t)-NF-κB-P65 expression among the four groups (Figure 7D and 7F). These results suggested that the TLR4-NF-κB pathways were involved in wogonin-mediated anti-inflammatory and neuro-protective effects in retina after ONC.

## DISCUSSION

A wide variety of animal models have been used to study the neuro-protective effects of a given treatment on the injured RGCs [31-33]. Upon ONC model, more than 90% of RGCs loss was within the first 2 weeks without concomitant death of other retinal neurons [34]. Thus, ONC is an ideal animal model to investigate RGCs survival and underlying mechanisms, and to explore the potential neuro-protective efficacy of a drug or compound in glaucoma [31]. In this study, we found that wogonin directly prevented RGCs death by inhibiting caspase-3-depandent apoptotic pathway. In addition, our results showed that wogonin could attenuate the release of pro-inflammatory cytokines, gliosis responses, and microglial activation in retina after ONC. We found that the neuro-protective and anti-inflammatory role of wogonin was able to *via* inhibiting the activation of TLR4-NF-κB pathways. Collectively, these findings provided the compelling evidences that wogonin possesses neuro-protective role in experimental glaucoma.

Wogonin, a group of flavonoid compounds, are known to possess a variety of biological effects, including anti-viral, anti-bacterial [19], anti-oxidant [19], anti-inflammatory [16], and anti-cancer [17] actions. Several studies have reported that wogonin protected neuronal death in oxidative stress-induced rat primary cortical neuron [35], and also significantly reduced neuronal death and attenuated brain edema in brain after TBI [27]. In addition, wogonin protected rat DRG neurons from Tunicamycin-induced apoptosis and ER stress as well [20, 21]. Furthermore, wogonin could protect retinal pigment epithelium cells from hydroperoxide-induced apoptosis [36]. These results suggested the potential anti-apoptotic effect of wogonin in the prevention of retinal diseases. Brn3a is a nuclear protein expressed exclusively in RGCs and has been widely used as a reliable and efficient marker to identify and quantify RGCs in normal and injured retina [13, 37]. In this work, we demonstrated that the number of Brn3a-positive RGCs and the Brn3a protein level were much lower in retina after ONC than those in control group, which were markedly increased after wogonin treatment, especially in the dose of 30 mg/kg. To further investigate the prevalence of apoptosis in RGCs, TUNEL staining was preformed to explore whether wogonin could reduce ONC-induced RGCs apoptosis. In control group, fewer TUNEL-positive cells were detected than those subjected to ONC. Compared to untreated ONC group, there were notably fewer TUNEL-positive cells in the GCL of retina after wogonin treatment. Thus, our current data demonstrated the anti-apoptotic effect of wogonin on RGCs in retina after ONC.

The molecular mechanism of RGCs death has been put to the effects of extrinsic and intrinsic apoptotic pathway [30, 38]. Both pathways are activated in apoptotic RGCs after animal models of ON injury. The mechanism of these two pathways on RGCs survival have been studied by several groups [30]. Specifically, the extrinsic pathway starts by the activation of death receptors, such as Fas, which then activates caspase-8, and in turn, activates downstream executioner caspases-3 [30]. The intrinsic pathway starts with the release of cytochrome *c* into the cytoplasm, inducing the formation of a complex, which binds and activates caspase-9, and then activates the effector caspase-3 [30]. Thus, the activation of caspase-3 is involved in both the extrinsic and the intrinsic apoptotic pathway [30]. A growing body of evidence demonstrates that caspase-3 activation in RGCs is up-regulated after ON injury [39-41], and inhibition of caspase-3 activation increase the survival of RGCs [28, 37]. In our present study, the increased expression of cleaved-caspase-3 in NeuN-positive RGCs suggested a possible activation of caspase-3-dependent pathway after ONC. However, wogonin treatment significantly inhibited activation of caspase-3. These results suggested that wogonin, by blocking caspase-3-mediated pro-apoptotic pathways, could prevent the apoptosis of RGCs in retina after ONC.

Astrocytes and Müller cells are the most common glial cells in the retina and play vital role in sustaining RGCs health [42]. In normal retina, glial cells clasp around blood vessels and connect them to neurons, and then supply neurons with nutrients [5, 43]. After ON injury, glia cells up-regulate GFAP expression and migrate into the injured retina to curtail damage or inflammatory response, and undergo a process of reactivation by forming astrogliosis that does not support axonal re-growth [42, 44]. The gliosis responses had been described in different retinal pathologies, including glaucoma [43, 44]. Our study provided evidence that the number of GFAP-positive fibers in the IPL was increased and the expression of GFAP protein was up-regulated in retina after ONC, which was significant inhibited after wogonin treatment. These results indicated that wogonin could inhibit the gliosis responses in astrocytes and Müller cells of retina after ONC.

Chronic neuro-inflammation is mainly conducted by the activation of microglial cells, the mainly immune surveillance cells in CNS and retina, and was also implicated in glaucoma [43]. In activated state after suffering from injuries such as ONC, “dendritic” microglial cells become “amoeboid” and exhibit enlarged somas and proliferative potential. Previous studies have reported that excessive and prolonged activation of microglia in retina has been associated with the loss of RGCs in glaucoma [45, 46]. This boosts the proposal that therapeutic strategies designed to reduce microglial reactivity may protect RGCs in glaucoma [45]. During activation, microglial cells are characterized by changes in cell morphology and up-regulated iba1 gene expression, leading to promote the release of pro-inflammatory cytokines [47]. Recent studies have shown that the microglial activation plays detrimental roles in glaucoma and inhibition of microglial activation protects RGCs death in several animal models of glaucoma [15, 45, 46]. It has been discovered that wogonin could inhibit the release of pro-inflammatory cytokines [23, 24] and reduce migration of LPS-induced microglia *in vitro* [25]. In addition, wogonin also significantly reduced microglial activation and the release of inflammatory cytokines in injured brain after TBI [27]. In our study, we found that wogonin significantly inhibited activation of microglial cells, which was manifested as a significant increase in the proportion of dendritic cells and decrease in the proportion of amoeboid cells. In addition, wogonin also reduced protein expression of iba1 and inhibited the release of inflammatory cytokines, such as TNF-α, MCP-1, iNOS, IL-6, IL-1β, and COX-2 in retina after ONC. These results indicated that the neuro-protective effects of wogonin in apoptotic RGCs might be related to inhibition of the microglial activation.

TLR4, an important signal transduction membrane protein, have been shown to play vital role in the innate immune system and the inflammatory responses [29]. On the surface of microglial cells, TLR4 activates several signal pathways, which leads to NF-κB activation in the end [29, 48]. In an inactive form, NF-κB bound to I-κB proteins, an interaction that regulates its activity in the cytoplasm. After multiple stimuli, I-κB proteins rapidly degrade and lead to the activation of NF-κB-P65, which then translocate into the nucleus, bind to specific DNA sites, and promote release of pro-inflammatory cytokines, resulting in the neuro-inflammation [29, 48]. Therefore, inhibiting TLR4-NF-κB-mediated release of pro-inflammatory cytokines caused by microglial activation would be an effective therapeutic approach to prevent the apoptotic RGCs in glaucoma. In our present study, we found that the protein level of TLR4 was increased in retina after ONC, and wogonin treatment inhibited TLR4 expression. In addition, NF-κB-P65 in nuclear fraction and phosphorylated NF-κB-P65 expression were increased in retina after ONC. Interestingly, wogonin treatment attenuated NF-κB-P65 phosphorylation, and down-regulated NF-κB-P65 DNA blinding in nuclear, suggesting that wogonin exerted its anti-inflammatory role *via* interfering with the TLR4-NF-κB pathways.

In conclusion, this study provided the compelling evidence that wogonin prevented apoptosis of RGCs by inhibiting the activation of caspase-3, reducing the release of pro-inflammatory cytokines, attenuating gliosis responses, and blocking microglial activation in retina after ONC. Furthermore, the neuro-protective and anti-inflammatory role of wogonin was exerted *via* inhibiting the activation of TLR4-NF-κB pathway. It is significant that the current investigation provides evidence that wogonin can be a potential therapy in the treatment of glaucoma.

## MATERIALS AND METHODS

### Animals

Adult male Sprague-Dawley rats (10 weeks old) weighing 250 g (220-275g) were used in this study. The rats obtained from the Animal Laboratory of Zhongshan Ophthalmic Center (Guangzhou, China) were housed on a 12 hours light/dark cycle and free accessed to food and water provided *ad libitum*. Animal care and experimental procedures were approved by the Institutional Animal Care and Use Committee of Zhongshan Ophthamic Center, and were performed in accordance with the ARVO Statement for the Use of Animals in Ophthalmic and Vision Research.

### ONC model

Only left optic nerve of the rats was crushed as described in previous studies [39, 49] with slight modification. Briefly, after general anesthesia induced by 10 % chloral hydrate, the ON was exposed and isolated after the gently rotated downward in the orbit. Considerable care was taken to avoid injury to the small vessels around the optic nerve. A vascular clip (60 g micro-vascular clip, World Precision Instruments, FL, USA) was used to clamp the optic nerve 2 mm posterior to the globe for 10 s. Before wound closure, the intactness of the retinal blood supply was verified upon ophthalmoscopy by checking the perfusion of the retinal vessels after ONC, and then Tobradex eye ointment (Alcon, Puurs, Belgium) was applied. Rats with severe reduction of the perfusion were excluded. Rats were placed in a heated cage to maintain body temperature while recovering from anesthesia. All animals were killed at different survival times (1, 7 or 14 days) after ONC.

### Wogonin preparation and administration

Wogonin (#CAS: 236-02321, Wako Pure Chemical Industries, Osaka, Japan) was administered intraperitoneally 10 min after establishment of ONC model. Wogonin was dissolved in 30% dimethyl sulfoxide (DMSO, 0.1 mL) and freshly prepared in different concentrations (0, 20, 30 or 40 mg/kg) according to the previous studies [26, 27]. After choosing the best concentration, rats were divided randomly into four groups: (1) control group: the age-matched mice kept in room air; (2) wogonin group: the age-matched mice kept in room air and intraperitoneal injection of 30 mg/kg wogonin solution; (3) ONC group: only suffering an ONC injury; (4) ONC-plus-wogonin group: ONC group and intraperitoneal injection of 30 mg/kg wogonin solution. In the ONC groups, no animals were lost before the determined time points. Total 144 rats were used for retinal flat mounting and Brn3a labeling (*n*: 6×6 = 36), Western blot analysis (*n*: 6×6 = 36), real time-PCR (*n*: 4×6 = 24), TUNEL assay and immunofluorescent staining (*n*: 4×6 = 24), and NF-κB activity analysis (*n*: 4×6 = 24).

### Retinal flat mounting, Brn3a labeling and counting

To evaluate the density of RGCs, retinal flat mounting and Brn3a labeling were performed according to previously published methods [50, 51]. The eyeballs were placed in 4 % paraformaldehyde (PFA) for 30 min and the whole retina was then carefully dissected and flattened. Then, retinas were dissected and blocked in phosphate buffered saline (PBS) containing 0.5 % Triton X-100 and 5 % bovine serum albumin (BSA) at 4 °C overnight. Subsequently, the retinas were incubated with goat polyclonal anti-Brn3a (1:50, RGCs marker, Cat. #C20, Santa Cruz) at 4 °C overnight. Retinas were washed with PBS 15 min for three times, and mounted on microscope slides. The retinas were examined for RGCs at a distance of 1-2 mm from the optic nerve head (ONH). We counted four areas, the size of each area was 225×170 μm^2^, including nasal superior, temperal superior, nasal inferior and temperal inferior. The regions of each retina were examined by confocal microscope (Zeiss510; CarlZeiss, Germany). The mean density of RGCs per retina was measure by the averages of these areas.

### Preparation of rat retina and immunofluorescent staining

Immunofluorescent staining was performed on 8 μm thick frozen sections as our previously described [39, 52]. All retinal sections were prepared at 1-2 mm distance from the ONH, which was to ensure the use of equivalent fields for comparison. Sections were blocked in Tris-buffered saline containing 0.2% Triton X-100 and 1% BSA for 1 h at room temperature to avoid unspecific staining. Then, sections were incubated overnight at 4 °C with primary antibodies for rabbit polyclonal anti-cleaved-caspase-3 (1:100 dilution, an apoptotic cells marker, Cat. #Asp175, Cell Signaling), mouse polyclonal anti-NeuN (1:100 dilution, a RGCs marker; Cat. #ABN78, Millipore), rabbit polyclonal anti-iba1 (1:200 dilution, a microglial cells marker; Cat. #019-19741, Wako), or rabbit monoclonal anti-GFAP (1:200 dilution, an astrocyte marker; Cat. #MAB3402, Millipore) at 4 °C overnight. After washing in PBS, a mixture of fluorescein isothiocyanate- and Cy3-conjugated secondary antibodies was added. DAPI was added to visualize the nuclear layers. The stained sections were examined with confocal microscope (Zeiss510; Carl Zeiss, Germany).

### TUNEL staining

TUNEL reactions was performed using the *In Situ* Cell Death Detection Kit that labels DNA strand breaks with fluorescein isothiocyanate (#CAS: 11684817910, Roche Molecular Biochemicals, Mannheim, Germany) to detect apoptotic RGCs as our previously described [52]. Frozen tissue sections rinsed in PBS and incubated for 60 min at 37 °C with 50 μl of TUNEL reaction mixture. DAPI was added to visualize the nuclear layers. After washing with PBS, the slides were analyzed by confocal microscope (Zeiss510; CarlZeiss, Germany).

### TUNEL-, GFAP- or iba1-positive cells counting analysis

To avoid counting the same cell in more than one section, we counted every fifth section (120 μm apart). The mean counts of TUNEL-positive cells in the GCL of each sample were counted at × 400 magnification, and three sections per eye were averaged. The GFAP-positive fibers in the astrocytes and Müller cells were quantified crossing the IPL and the inner nuclear layer (INL). The mean counts of GFAP- or iba1-positive cells in the whole retina of each sample were counted at × 400 magnification, and three sections per eye were averaged. According to whether the cells displayed dendritic or amoeboid morphology, the counts of total number of microglial cells was subdivided. A small number of iba1-positive cells were omitted from the counts, if they could not be determined on the basis of their morphology. Cell counting was performed by two independent masked investigators (XY & YB) and with a computerized image-analysis system (Image Pro Plus Version 6.0; Media Cybernetics, Silver Spring, MD) in duplicate.

### Protein extraction and western blot analyses

Western blot analyses were performed as our previously described [39, 52]. 20-50 μg protein was separated with SDS-PAGE and transferred to PVDF membrane (Millipore, Bedford, MA). After blocking with 5% defatted milk in PBS-Tween-20 for 1 h at room temperature, the PDF membrane was incubated with incubated with primary antibodies for goat polyclonal anti-Brn3a (1:500 dilution, Cat. #C20, Santa Cruz), mouse polyclonal anti-Cleaved-caspase-3 (1:600 dilution, Cat. #Asp175, Cell Signaling), rabbit polyclonal anti-iba1 (1:500 dilution, Cat. #016-20001, Wako), or rabbit monoclonal anti-GFAP (1:1000 dilution, Cat. #MAB3402, Millipore), rabbit polyclonal anti-phosphorylated (p)-NF-κB-P65 (1:300 dilution, Cat. #sc-136548, Santa Cruz), rabbit polyclonal anti-total (t)-NF-κB-P65 (1:500 dilution, Cat. #ab16502, abcam), mouse monoclonal anti-TLR4 (1:500 dilution, Cat. #ab22048, abcam), and mouse monoclonal anti-β-actin (1:1000 dilution, Cat. #ab8226, abcam) at 4°C overnight. After washed with PBS-Tween-20 every 15 min for three times, the membrane was incubated with goat-anti-rabbit or goat-anti-mouse second antibody conjugated horseradish peroxidase (1: 10,000; Abgent) for 2 h and then scanned with the Odyssey infrared imaging system (LI-COR Bioscience).

### Real time PCR

After group-specified experimental conditions, total RNA of retinas was extracted using Trizol and converted to cDNA by using a cDNA first-strand synthesis system (Fermentas, Canada). The PCR primers were designed based on the NCBI mRNA and genome DNA sequence database. The primers of target genes were as follows: TNF-α forward primer: CGT GGA ACT GGC AGA AGA GG and reverse primer: CTG CCA CAA GCA GGA ATG AG. MCP-1 forward primer: 5′-3′-ACT GAA GCC AGC TCT CTC TTC CTC and reverse primer: 5′-3′-TTC CTT CTT GGG GTC AGC ACA GAC; iNOS forward primer: 5′-3′-ACA ACA GGA ACC TAC CAG CTC A and reverse primer: 5′-3′-GAT GTT GTA GCG CCT GTG TGT CA; IL-6 forward primer: CCA GAA ACC GCT ATG AAG TTC C and reverse primer: GTT GGG AGT GGT ATC CTC TGT GA; IL-1β forward primer: GTT CCC ATT AGA CAA CTG CAC TAC AG and reverse primer: GTC GTT GCT TGG TTC TCC TTG TA; COX-2 forward primer: 5′-3′-CCA GAT GAT ATC TTT GGG GAG AC and reverse primer: 5′-3′-CTT GCA TTG ATG GTG GCT G; β-actin forward primer: 5′-3′-GGC GGA CTA TGA CTT AGT TG and reverse primer: 5′-3′-AAA CAA CAA TGT GCA ATC AA. DNA fragments of PCR products were designed to amplify within 200bp length. Results were normalized from β-actin of respective samples. Briefly, each reaction contained 0.8 μl primer (containing 100 nM forward and reverse primers), 2 μl of cDNA (0.1 μl of RNA equivalent), 5 μl of Sofast EvaGreen supermix and 2.2 μl of H_2_O. Real Time-PCR was performed at 94 °C for 45 s, and 55 °C for 45 s to denaturation, and at 72 °C for 45 s for 50 cycles to extension. All data were analyzed by CFX manager software (BioRad, Hercules, CA, USA).

### Nuclear extraction and NF-κB activity analysis

After group-specified experimental conditions, nuclear extracts from retina were prepared using the Nuclear Extract Kit (CAS. #40010, Active Motif, Carlsbad, CA) following the manufacturer's instructions. NF-κB activity was measured by a NF-κB p65 Transcription Factor Assay Kit (CAS. #10007889, Cayman Chemical, Shanghai, China) according to the manufacturer's protocol.

### Statistical analysis

All measurements were performed in a blinded fashion. Data are presented as mean ± SEM. Statistical analyses were performed with GraphPad Prism (v6.0) (GraphPad Software Inc.). In all cases, *p* < 0.05 was considered statistically significant. Two-way ANOVA followed by Turkey multiple comparison was used when different groups were compared.

## References

[1] Wan P, Su W, Zhuo Y (2016). The Role of Long Noncoding RNAs in Neurodegenerative Diseases. Molecular neurobiology.

[2] Nafissi N, Foldvari M (2015). Neuro-protective therapies in glaucoma: II. Genetic nanotechnology tools. Frontiers in neuroscience.

[3] Nafissi N, Foldvari M (2016). Neuro-protective therapies in glaucoma: I. Neurotrophic factor delivery. Wiley interdisciplinary reviews Nanomedicine and nanobiotechnology.

[4] Doozandeh A, Yazdani S (2016). Neuroprotection in Glaucoma. Journal of ophthalmic & vision research.

[5] Tian K, Shibata-Germanos S, Pahlitzsch M, Cordeiro MF (2015). Current perspective of neuroprotection and glaucoma. Clinical ophthalmology.

[6] Wong M, Li Y, Li S, Zhang S, Li W, Zhang P, Chen C, Barnstable CJ, Zhang SS, Zhang C, Huang P (2015). Therapeutic Retrobulbar Inhibition of STAT3 Protects Ischemic Retina Ganglion Cells. Molecular neurobiology.

[7] Mac Nair CE, Nickells RW (2015). Neuroinflammation in Glaucoma and Optic Nerve Damage. Progress in molecular biology and translational science.

[8] Sapienza A, Raveu AL, Reboussin E, Roubeix C, Boucher C, Degardin J, Godefroy D, Rostene W, Reaux-Le Goazigo A, Baudouin C, Melik Parsadaniantz S (2016). Bilateral neuroinflammatory processes in visual pathways induced by unilateral ocular hypertension in the rat. Journal of neuroinflammation.

[9] Jindal V (2015). Interconnection between brain and retinal neurodegenerations. Molecular neurobiology.

[10] Bond WS, Rex TS (2014). Evidence That Erythropoietin Modulates Neuroinflammation through Differential Action on Neurons, Astrocytes, and Microglia. Frontiers in immunology.

[11] Lin S, Liang Y, Zhang J, Bian C, Zhou H, Guo Q, Xiong Y, Li S, Su B (2012). Microglial TIR-domain-containing adapter-inducing interferon-beta (TRIF) deficiency promotes retinal ganglion cell survival and axon regeneration via nuclear factor-kappaB. Journal of neuroinflammation.

[12] Halder SK, Matsunaga H, Ishii KJ, Ueda H (2015). Prothymosin-alpha preconditioning activates TLR4-TRIF signaling to induce protection of ischemic retina. Journal of neurochemistry.

[13] Morzaev D, Nicholson JD, Caspi T, Weiss S, Hochhauser E, Goldenberg-Cohen N (2015). Toll-like receptor-4 knockout mice are more resistant to optic nerve crush damage than wild-type mice. Clinical & experimental ophthalmology.

[14] Okamoto T, Ozawa Y, Kamoshita M, Osada H, Toda E, Kurihara T, Nagai N, Umezawa K, Tsubota K (2016). The Neuro-protective Effect of Rapamycin as a Modulator of the mTOR-NF-kappaB Axis during Retinal Inflammation. PloS one.

[15] Ulbrich F, Schallner N, Coburn M, Loop T, Lagreze WA, Biermann J, Goebel U (2014). Argon inhalation attenuates retinal apoptosis after ischemia/reperfusion injury in a time- and dose-dependent manner in rats. PloS one.

[16] Lucas CD, Dorward DA, Sharma S, Rennie J, Felton JM, Alessandri AL, Duffin R, Schwarze J, Haslett C, Rossi AG (2015). Wogonin induces eosinophil apoptosis and attenuates allergic airway inflammation. American journal of respiratory and critical care medicine.

[17] Chirumbolo S (2013). Anticancer properties of the flavone wogonin. Toxicology.

[18] Lin B (2011). Polyphenols and neuroprotection against ischemia and neurodegeneration. Mini reviews in medicinal chemistry.

[19] Gasiorowski K, Lamer-Zarawska E, Leszek J, Parvathaneni K, Yendluri BB, Blach-Olszewska Z, Aliev G (2011). Flavones from root of Scutellaria baicalensis Georgi: drugs of the future in neurodegeneration?. CNS & neurological disorders drug targets.

[20] Chen F, Wu R, Zhu Z, Yin W, Xiong M, Sun J, Ni M, Cai G, Zhang X (2015). Wogonin protects rat dorsal root ganglion neurons against tunicamycin-induced ER stress through the PERK-eIF2alpha-ATF4 signaling pathway. Journal of molecular neuroscience.

[21] Xu S, Zhao X, Zhao Q, Zheng Q, Fang Z, Yang X, Wang H, Liu P, Xu H (2015). Wogonin prevents rat dorsal root ganglion neurons death via inhibiting tunicamycin-induced ER stress in vitro. Cellular and molecular neurobiology.

[22] Chen S, Xiong J, Zhan Y, Liu W, Wang X (2015). Wogonin inhibits LPS-induced inflammatory responses in rat dorsal root ganglion neurons via inhibiting TLR4-MyD88-TAK1-mediated NF-kappaB and MAPK signaling pathway. Cellular and molecular neurobiology.

[23] Yeh CH, Yang ML, Lee CY, Yang CP, Li YC, Chen CJ, Kuan YH (2014). Wogonin attenuates endotoxin-induced prostaglandin E2 and nitric oxide production via Src-ERK1/2-NFkappaB pathway in BV-2 microglial cells. Environmental toxicology.

[24] Yeh CH, Shih HC, Hong HM, Lee SS, Yang ML, Chen CJ, Kuan YH (2015). Protective effect of wogonin on proinflammatory cytokine generation via Jak1/3-STAT1/3 pathway in lipopolysaccharide stimulated BV2 microglial cells. Toxicology and industrial health.

[25] Piao HZ, Choi IY, Park JS, Kim HS, Cheong JH, Son KH, Jeon SJ, Ko KH, Kim WK (2008). Wogonin inhibits microglial cell migration via suppression of nuclear factor-kappa B activity. International immunopharmacology.

[26] Cho J, Lee HK (2004). Wogonin inhibits ischemic brain injury in a rat model of permanent middle cerebral artery occlusion. Biological & pharmaceutical bulletin.

[27] Chen CC, Hung TH, Wang YH, Lin CW, Wang PY, Lee CY, Chen SF (2012). Wogonin improves histological and functional outcomes, and reduces activation of TLR4/NF-kappaB signaling after experimental traumatic brain injury. PloS one.

[28] Liu Y, Yan H, Chen S, Sabel BA (2015). Caspase-3 inhibitor Z-DEVD-FMK enhances retinal ganglion cell survival and vision restoration after rabbit traumatic optic nerve injury. Restorative neurology and neuroscience.

[29] Shabab T, Khanabdali R, Moghadamtousi SZ, Kadir HA, Mohan G (2016). Neuroinflammation Pathways: a General Review. The International journal of neuroscience.

[30] Almasieh M, Wilson AM, Morquette B, Cueva Vargas JL, Di Polo A (2012). The molecular basis of retinal ganglion cell death in glaucoma. Progress in retinal and eye research.

[31] McKinnon SJ, Schlamp CL, Nickells RW (2009). Mouse models of retinal ganglion cell death and glaucoma. Experimental eye research.

[32] Rathnasamy G, Foulds WS, Ling EA, Kaur C (2016). Glutamate Inhibits the Pro-Survival Effects of Insulin-Like Growth Factor-1 on Retinal Ganglion Cells in Hypoxic Neonatal Rat Retina. Molecular neurobiology.

[33] Ribas VT, Koch JC, Michel U, Bahr M, Lingor P (2016). Attenuation of Axonal Degeneration by Calcium Channel Inhibitors Improves Retinal Ganglion Cell Survival and Regeneration After Optic Nerve Crush. Molecular neurobiology.

[34] Morgan-Warren PJ, O'Neill J, de Cogan F, Spivak I, Ashush H, Kalinski H, Ahmed Z, Berry M, Feinstein E, Scott RA, Logan A (2016). siRNA-Mediated Knockdown of the mTOR Inhibitor RTP801 Promotes Retinal Ganglion Cell Survival and Axon Elongation by Direct and Indirect Mechanisms. Investigative ophthalmology & visual science.

[35] Cho J, Lee HK (2004). Wogonin inhibits excitotoxic and oxidative neuronal damage in primary cultured rat cortical cells. European journal of pharmacology.

[36] Yan T, Bi H, Wang Y (2014). Wogonin modulates hydroperoxide-induced apoptosis via PI3K/Akt pathway in retinal pigment epithelium cells. Diagnostic pathology.

[37] Sanchez-Migallon MC, Valiente-Soriano FJ, Nadal-Nicolas FM, Vidal-Sanz M, Agudo-Barriuso M (2016). Apoptotic Retinal Ganglion Cell Death After Optic Nerve Transection or Crush in Mice: Delayed RGC Loss With BDNF or a Caspase 3 Inhibitor. Investigative ophthalmology & visual science.

[38] Chen SD, Wang L, Zhang XL (2013). Neuroprotection in glaucoma: present and future. Chinese medical journal.

[39] Huang Y, Xu Y, Cheng Q, Yu S, Gao Y, Shu Q, Yang C, Sun Y, Wang J, Xu F, Liang X (2014). The expression changes of myelin and lymphocyte protein (MAL) following optic nerve crush in adult rats retinal ganglion cells. Journal of molecular neuroscience.

[40] Zhang G, Han M, Wang X, Xiao A (2015). GRP75 Involves in Retinal Ganglion Cell Apoptosis After Rat Optic Nerve Crush. Journal of molecular neuroscience.

[41] Xu F, Huang H, Wu Y, Lu L, Jiang L, Chen L, Zeng S, Li L, Li M (2014). Upregulation of Gem relates to retinal ganglion cells apoptosis after optic nerve crush in adult rats. Journal of molecular histology.

[42] Spaide RF (2016). Retinal vascular cystoid macular edema: Review and New Theory. Retina.

[43] Ramirez AI, Salazar JJ, de Hoz R, Rojas B, Gallego BI, Salobrar-Garcia E, Valiente-Soriano FJ, Trivino A, Ramirez JM (2015). Macro- and microglial responses in the fellow eyes contralateral to glaucomatous eyes. Progress in brain research.

[44] de Hoz R, Rojas B, Ramirez AI, Salazar JJ, Gallego BI, Trivino A, Ramirez JM (2016). Retinal Macroglial Responses in Health and Disease. BioMed research international.

[45] Madeira MH, Boia R, Elvas F, Martins T, Cunha RA, Ambrosio AF, Santiago AR (2016). Selective A2A receptor antagonist prevents microglia-mediated neuroinflammation and protects retinal ganglion cells from high intraocular pressure-induced transient ischemic injury. Translational research.

[46] Wilson GN, Inman DM, Dengler Crish CM, Smith MA, Crish SD (2015). Early pro-inflammatory cytokine elevations in the DBA/2J mouse model of glaucoma. Journal of neuroinflammation.

[47] Hong H, Kim BS, Im HI (2016). Pathophysiological Role of Neuroinflammation in Neurodegenerative Diseases and Psychiatric Disorders. International neurourology journal.

[48] Jiang L, Xu F, He W, Chen L, Zhong H, Wu Y, Zeng S, Li L, Li M (2016). CD200Fc reduces TLR4-mediated inflammatory responses in LPS-induced rat primary microglial cells via inhibition of the NF-kappaB pathway. Inflammation research.

[49] Zhong H, Cui L, Xu F, Chen L, Jiang L, Huang H, Xu J, Zhao X, Li L, Zeng S, Li M (2016). Up-regulation of Wip1 involves in neuroinflammation of retinal astrocytes after optic nerve crush via NF-kappaB signaling pathway. Inflammation research.

[50] Nadal-Nicolas FM, Rodriguez-Villagra E, Bravo-Osuna I, Sobrado-Calvo P, Molina-Martinez I, Villegas-Perez MP, Vidal-Sanz M, Agudo-Barriuso M, Herrero-Vanrell R (2016). Ketorolac Administration Attenuates Retinal Ganglion Cell Death After Axonal Injury. Investigative ophthalmology & visual science.

[51] Galindo-Romero C, Valiente-Soriano FJ, Jimenez-Lopez M, Garcia-Ayuso D, Villegas-Perez MP, Vidal-Sanz M, Agudo-Barriuso M (2013). Effect of brain-derived neurotrophic factor on mouse axotomized retinal ganglion cells and phagocytic microglia. Investigative ophthalmology & visual science.

[52] Xu Y, Yu S, Shu Q, Yang L, Yang C, Wang J, Xu F, Ji M, Liang X (2014). Upregulation of CREM-1 relates to retinal ganglion cells apoptosis after light-induced damage in vivo. Journal of molecular neuroscience.

